# PW01-010 – The effect of pregnancy on disease course in FMF

**DOI:** 10.1186/1546-0096-11-S1-A63

**Published:** 2013-11-08

**Authors:** T Eviatar, N Zaks, OL Kukuy, A Livneh, M Lidar

**Affiliations:** 1 Sheba Medical Center, Ramat Gan, Israel

## Introduction

FMF is the most common of the hereditary periodic fever syndromes. It usually begins in the first two decades of life, and as such, has a significant impact on women in their reproductive years. To date, only one retrospective study, investigating the course of FMF in pregnancy, has been published.

## Objectives

To examine the course of disease in FMF patients, while pregnant, and to evaluate the association of pregnancy with attack frequency and severity.

## Methods

All pregnant FMF patients treated at the FMF clinic of the Sheba Medical Center from May 2010 onwards, consenting to participate in this observational study, were studied prospectively for the occurrence of attacks. Attacks were recorded by patients in diaries provided on enrollment and relied by phone to a study coordinator on a monthly telephone call or on a pre-specified physician visit, whichever occurred first. In addition to noting attack occurrence, patients were instructed to record attack severity (on a 1-10 scale), attack location, duration, medications taken during the attack, colchicine dose prior to and during the attack, as well as ancillary signs and symptoms related to their FMF such as the occurrence of exertional leg pain.

## Results

We present results of 28 pregnancies in 24 patients. Average attack rate remained unchanged throughout the pregnancy and the 9 month follow-up period post delivery (Average attack rates per 3 months, in the year before pregnancy vs. 1^st^, 2^nd^ and 3^rd^ trimester of pregnancy as well as the 9 months post delivery are 2.42 95%CI: 1.3-6.1, 2.4 95%CI:0.8-4, 2.2 95%CI:0.9-2.5, 2.2 95%CI:1.1-4.3 and 1.94 95%CI 0.2-3.6, respectively).That being, an amelioration in attack rate was experienced in 25% of the pregnancies including 10.7% of pregnancies in which attack rate decreased by more than 80% compared to the pre-pregnancy value. Conversely, in 28.5% of pregnancies patients experienced more frequent attacks. No significant changes in attack severity or location, nor in colchicine dose were noted. Early delivery was indicated in about 10% of cases including a single patient with pre-existing proteinuria, and increasing urinary protein levels during the 3^rd^ trimester. Pregnancy outcome was favorable in all cases.

## Conclusion

Pregnancy in FMF is safe for patients and their offspring. Odds for amelioration, worsening or endurance of prior course are similarly shared.

## Disclosure of interest

None declared

